# SERCA2 Regulates Non-CF and CF Airway Epithelial Cell Response to Ozone

**DOI:** 10.1371/journal.pone.0027451

**Published:** 2011-11-11

**Authors:** Shama Ahmad, David P. Nichols, Matthew Strand, Raymond C. Rancourt, Scott H. Randell, Carl W. White, Aftab Ahmad

**Affiliations:** 1 Department of Pediatrics, National Jewish Health, Denver, Colorado, United States of America; 2 Division of Biostatistics and Bioinformatics, National Jewish Health, Denver, Colorado, United States of America; 3 Cystic Fibrosis/Pulmonary Research and Treatment Center, Department of Medicine, The University of North Carolina at Chapel Hill, Chapel Hill, North Carolina, United States of America; University of Pittsburgh, United States of America

## Abstract

Calcium mobilization can regulate a wide range of essential functions of respiratory epithelium, including ion transport, ciliary beat frequency, and secretion of mucus, all of which are modified in cystic fibrosis (CF). SERCA2, an important controller of calcium signaling, is deficient in CF epithelium. We conducted this study to determine whether SERCA2 deficiency can modulate airway epithelial responses to environmental oxidants such as ozone. This could contribute to the pathogenesis of pulmonary exacerbations, which are important and frequent clinical events in CF. To address this, we used air-liquid interface (ALI) cultures of non-CF and CF cell lines, as well as differentiated cultures of cells derived from non-CF and CF patients. We found that ozone exposure caused enhanced membrane damage, mitochondrial dysfunction and apoptotic cell death in CF airway epithelial cell lines relative to non-CF. Ozone exposure caused increased proinflammatory cytokine production in CF airway epithelial cell lines. Elevated proinflammatory cytokine production also was observed in shRNA-mediated SERCA2 knockdown cells. Overexpression of SERCA2 reversed ozone-induced proinflammatory cytokine production. Ozone-induced proinflammatory cytokine production was NF-κB- dependent. In a stable NF-κB reporter cell line, SERCA2 inhibition and knockdown both upregulated cytomix-induced NF-κB activity, indicating importance of SERCA2 in modulating NF-κB activity. In this system, increased NF-κB activity was also accompanied by increased IL-8 production. Ozone also induced NF-κB activity and IL-8 release, an effect that was greater in SERCA2-silenced NF-κB-reporter cells. SERCA2 overexpression reversed cytomix-induced increased IL-8 release and total nuclear p65 in CFTR-deficient (16HBE-AS) cells. These studies suggest that SERCA2 is an important regulator of the proinflammatory response of airway epithelial cells and could be a potential therapeutic target.

## Introduction

Atmospheric pollutants such as ozone, particulates and nitrogen oxides continuously challenge airways of urban dwellers. Elevated pollutant levels may contribute to exacerbations, and accelerated decline of lung function, in patients with chronic airway disease like cystic fibrosis (CF) and asthma [1], [2], [3]. Ozone attacks the lung through oxidative mechanisms, causing disruption of epithelial barrier, increased permeability, influx of neutrophils and generation of cytokines and chemokines [4], [5], [6], [7]. We have previously established the role of ozone-reactive surfactant phospholipids in modulating airway epithelial cell viability in response to ozone [8]. These derivatives not only caused apoptotic cell death but also induced proinflammatory responses that may potentiate airway injury in asthmatics and other susceptible patients like those with cystic fibrosis (CF). Increased oxidative stress and enhanced lipid derived inflammatory mediators are characteristic of CF patient biofluids, during respiratory exacerbations [9]. Acute exacerbations accelerate the clinical progression of CF and hasten decline of lung function. Contribution of environmental ozone to exacerbations in CF symptoms has been suggested [1]. However, identification of mechanisms leading to pulmonary exacerbations in patients with CF is crucial for developing therapies for maintenance of lung function, quality of life and survival.

We have recently established that in CF airway epithelium there is decreased expression of an important ER calcium pump, sarcoendoplasmic reticulum calcium ATPase, SERCA2 [10]. We have also documented a role for SERCA2 in survival of airway epithelium in oxidative stress, including that caused by ozone [10]. SERCA2 is the only pump that actively loads Ca^2+^ back into ER of airway cells for subsequent signaling events once it has been released following IP3 receptor activation on ER. Therefore, abnormalities in this enzyme could have critical consequences. This is evident in SERCA2 gene knockout studies, where mice do not survive to birth [11]. One obvious consequence of decreased activity of this pump would be a sustained increase in cytosolic calcium resulting in passive overloading of cellular calcium stores. Expanded or overloaded calcium stores have been demonstrated by previous studies in cystic fibrosis airway epithelial cells [12], [13]. Sustained increase in cytosolic calcium has also been shown to upregulate expression of proinflammatory transcriptional regulators such as NF-κB [14]. Similarly, inhibition of SERCA2 by specific inhibitor thapsigargin and subsequent increase in cytosolic calcium has been shown to potentiate NF-κB activation and IL-8 release response in airway epithelial cells [15], [16]. In this study we demonstrate that ozone exposure causes an increased proinflammatory response in CF airway epithelial cells. This enhanced proinflammatory response could be mediated by decreased SERCA2 that induces NF-κB promoter activity. These studies provide insight into the mechanism of noxious inflammatory response in CF, Supporting studies also provide evidence that SERCA2 could regulate NF-κB and that modulation of SERCA2 could be a potential therapeutic strategy for treatment of CF.

## Materials and Methods

### Cell culture

Human lung tissue was procured from National Disease Research Interchange (NDRI) under University of North Carolina (UNC) and National Jewish Institutional Review Board (NJIRB)-approved protocols and human bronchial epithelial cell harvest and culture was performed using established procedures previously described in detail [17]. Epithelial cells were removed from lower trachea and bronchi by protease XIV digestion and cells were plated in BEGM medium on collagen-coated dishes as described previously [10], [17]. Primary epithelial cells (passage 2; 5×10^5^ cells) were seeded onto 12 mm diameter type IV collagen (Sigma) coated Snapwells (0.4 µM pore size, Corning, Corning, NY) and following confluence on day 4-5 were maintained at an air-liquid interface (ALI). Experiments comparing ozone-induced proinflammatory cytokine release in primary non-CF and CF cells (demographics of donors provided in Table 1) were performed with differentiated air-liquid-interface (ALI) cultures grown simultaneously and matched for passage number, number of cells plated, and days in culture. Transformed human CF airway epithelia cell lines, CFBE41o- (CF41o-) and CFBE45o- (CF45o-) and a wild-type, 16HBE14o- (16HBEo-), were provided by Prof. D. Gruenert (California Pacific Medical Center Research Institute, University of California at San Francisco). These cell lines were cultured in Eagle's minimal essential medium (Invitrogen, Carlsbad, CA) supplemented with 10% fetal bovine serum, L-glutamine, and penicillin/streptomycin at 37°C under 5% CO_2_. Human bronchial epithelial 16HBEo- cells with stable expression of sense (16HBE-S) and antisense CFTR (16HBE-AS) oligonucleotides were provided by Dr. Pamela Davis, Case Western Reserve University Cleveland, OH and cultured as described previously [10]. Trans-epithelial resistance (TER) was also measured using previously described procedure [18].

**Table 1:** Demographics of airway tissue donors for epithelial cells.

Donor No.	Category	Age (yr)/Sex	COD*	Genotype
1	NTD	29/M	Head Trauma	Non-CF
2	NTD	30/M	MVA/Head Trauma	Non-CF
3	TD	22/F	Head Trauma	Non-CF
4	CF transplant	22/F		ΔF508/ΔF508
5	CF transplant	27/M		ΔF508/?
6	CF transplant	29/F		ΔF508/ΔF508

*COD, Cause of death, NTD =  non transplant donor, TD =  excess tissue from transplant donor.

MVA = motor vehicle accident. ? =  mutation not identified.

### Ozone exposures

Exposure of airway epithelial cells to ozone at precise levels was carried out in a computer- controlled *in vitro* exposure chamber [18]. Differentiated primary airway epithelial cells that were exposed to ozone contained a very thin layer of media apically. Cells were rocked so as to expose one side of apical surface at a time to ozone.

### Determination of cell survival and protein concentration

To assess cell death Calcein-AM and Propidium iodide were added apically on the inserts after treatment. Caspase 3/7 activity was measured by using Caspase-Glo^R^ 3/7-assay kit (Promega Corporation, Madison, WI). TUNEL staining on the inserts was performed using ApopTag^R^ Plus Peroxidase *In Situ* apoptosis kit (Millipore, Billerica, MA). Annexin binding was assessed by using Vybrant apotosis assay kit (Molecular Probes, Eugene, OR), as described before [18]. In this method, apoptotic cells bearing phosphatidylserine in the plasma membrane outer leaflet were identified as those binding Alexa Fluor 488-labeled annexin V. Mitochondrial membrane potential (MMP) determination was performed as described before [19]. Protein concentration in cell lysates was determined using the Bio-Rad DC protein assay kit (Bio-Rad, Hercules, Ca) in a 96-well plate with bovine serum albumin as a standard.

### Western blot

Western blots were performed as previously described in detail [20] and the membranes were probed with rabbit polyclonal antibodies against SERCA2 (Affinity Bioreagents, Golden, CO) at 1∶1,000 dilution for each, overnight at 4°C. Blots were then washed again with TBS-T and incubated with secondary peroxidase-conjugated IgG (Bio-Rad, Hercules, CA) at 1∶2,000 dilution, for 1 h at room temperature. Immunoreactive bands were detected using an ECL detection kit (Pierce, Rockford, IL) followed by exposure to Hyperfilm (Amersham Pharmacia Biotech Inc. UK). Antibody against cytochrome c (Affinity Bioreagents, Golden, CO) was used at a dilution of 1∶1000.

### Immunofluorescence staining

Cells grown on glass coverslips in 6-well plates were fixed in 4% paraformaldehyde (PFA) for 10 minutes, rinsed in TBS and permeabilized with 0.4% Triton-X-100 in 10 mM sodium citrate for 20 minutes. After blocking in 5% donkey serum for 20 minutes, cells were incubated with rabbit anti-cytochrome c (Affinity Bioreagents, Golden, CO) for 1 hour. Negative controls included normal rabbit IgG at same concentration as the primary antibodies used. A secondary antibody, anti-rabbit or FITC-conjugated donkey anti-mouse was then applied for 60 minutes. Cells were mounted on slides with Prolong Gold-DAPI and allowed to dry overnight. Slides were viewed using a Zeiss, Axiovert 200M fluorescent microscope and digital images recorded using Slidebook software (Intelligent Imaging Innovations, Denver, CO).

### Cytokine and total p65 assay

Apical and basolateral media was collected, centrifuged and supernatant was analyzed for cytokines by ELISA at ELISATech (Denver, CO). Nuclear lysates were analyzed for total p65 using NF-κB p65 (Total) Elisa kit (Invitrogen, Carlsbad, CA). NF-κB inhibitor [6-amino-4- (4-phenoxyphenylethylamino) quinazoline] was obtained from EMD Biosciences La Jolla CA.

### Respiratory mucin analysis

Human airway epithelial cell cultures were analyzed for extracellular levels of respiratory mucins by use of a double-sandwich ELISA system (MPY-661P) from Covance (Princeton, NJ) [21]. The cell surface was washed with PBS and incubated with DTT (6 mM) in PBS for 30 min. Supernatants were carefully collected using a 1.0 ml pipette tip and stored at −20°C for subsequent analysis. A delay of 24 h was deliberately used to ensure oxidation of DTT and minimize its effect in assays. For mucin analysis, 96-well plates were coated with purified monoclonal mucin antibody (17B1, Covance, Princeton, NJ) (0.3 µg/well) in 100 µl of coating buffer (0.05 M sodium carbonate buffer, pH 9.6) for 3 h under airtight cover. Wells were then rinsed 3 times using 150 µl of PBS-Tween 20 (0.05%). Samples were diluted in PBS-Tween (0.025%) and added to wells and the plates were sealed and allowed to incubate at 37°C for 4 h. Wells were rinsed 3 times and then reacted with 100 µl of PBS-tween 20 containing 0.4 µg of the alkaline phosphatase-conjugated 17Q2 antibody. Plates were then incubated overnight at 4°C on a rotary shaker set to 50 rpm. Fluids were removed and each well rinsed 3 times and then filled with 100 µl of substrate solution (1 mg/ml ρ-nitrophenyl phosphate disodium in 10% diethanolamine; pH 9.8). Absorbance change was monitored at 405 nm wavelength using a microplate reader. A standard curve was prepared using a stock of respiratory mucins, the protein concentration of which was determined using the optical method of Warburg and Christian [22].

### Lentiviral transduction

Lentivirus each carrying 5 different SERCA2 target shRNA (pLKO.1-CMV-tGFP-ATP2A2) and controls (pLKO.1-CMVtGFP-TurboGFP control) were obtained from Sigma (St.Louis MO). Transductions were carried out using standard procedure and according to manufacturer's instructions and SERCA2 knock down was tested using real time RTPCR. The shRNA sequence giving more than 50% SERCA2 knockdown was used.

### Adenoviral transduction

Transduction of SERCA2 and GFP-encoding adenoviral vectors was carried out as described before [23]. Ad.SERCA2 was a kind gift of Dr. R. J. Hajjar, Harvard Medical School, Massachusetts General Hospital, Cardiovascular Research Center, Charlestown, MA [24]. Recombinant viruses were added to cell cultures (multiplicity of infection, MOI 10∶1) on day 3 of culture, with exposure for 17 hours duration. Transduction efficiency was estimated by observing green fluorescence of adenoviral GFP-transduced cells.

### Stable NF-κB reporter cell lines construction

Reporter cell lines were generated by stably transducing 16HBEo- cells with GreenFire1™ pGF1-NF-κB reporter lentiviral vector (Systems Biosciences, Mount View, CA). This vector is a Human Immunodeficiency Virus (HIV) lentiviral vector that expresses destabilized (ds) copGFP reporter and firefly luciferase under the control of four NF-κB response elements and a minimal CMV promoter. The transfected cells were treated with TNFα (10 ng/ml) overnight then sorted using Fluorescence Activated Cell Sorting (FACS) using MoFlo Cell Sorter (Dako Cytomation, Carpinteria, CA) for high GFP expression. Sorted cells were cultured and sorted again after TNF treatment and individual clones were isolated and cultured.

### SERCA2 gene silencing using siRNA and luciferase reporter assay

SERCA2 gene silencing was carried out as described previously [10]. Briefly, predesigned human ‘SMARTPOOL’ SERCA2 siRNAs were purchased from Dharmacon (Lafayette, CO). NF-κB reporter expressing 16HBEo- cell lines were cultured on fibronectin-coated 6-well plates and transfected with 50 nM siRNA using DharmaFECT2 siRNA transfection reagent (Dharmacon; Lafayette, CO) according to manufacturer's instructions as described before [10]. Silencer negative control siRNA was used as a nonspecific siRNA, and mock transfection was used as a negative control. Cells were treated 48 h post-transfection and cell lysates were made using Reporter Lysis Buffer (Promega, Madison WI). Luciferase activities were determined with equal amounts of protein by using a commercially available luciferase assay system (BD Pharmingen, San Jose, CA) and a Monolight 3010 luminometer (Analytical Luminescence Laboratory, San Diego, CA).

### Statistical analysis

All statistical calculations were performed with JMP and SAS software (SAS Institute, Cary, NC). Data from experiments using one cell line per disease group were analyzed using two-way analysis of variance (ANOVA), with ozone level and disease (or treatment) category as the two factors, including the interaction term. For experiments involving multiple subjects per disease group (see section entitled ‘*Ozone-dependent cytokine release in differentiated primary non-CF and CF epithelial cells’* in Results) a linear mixed model was fit for each cytokine and media combination, using ozone level, disease category, and their interaction as predictors; a random intercept term for subjects was also included to account for repeated measures within subjects across factor levels.

Generally, pairwise comparisons were performed using t-tests, comparing the given ozone level to no ozone within a disease or treatment category and between a given disease or treatment category to no disease (or reference category) within a fixed level of ozone. Variables with right-skewed distributions were analyzed on the natural log scale in order to better meet model assumptions. The log transformation was also used for data with multiple subjects in order to stabilize variances between ozone*disease groups. Graphs were constructed based on untransformed sample data.

## Results

Exposure to ozone causes acute toxicity to human airways. Using non-polarized 16HBEo- cell lines we have demonstrated previously that exposure to ozone causes enhanced cell death [18]. We have also demonstrated that the airway epithelial cells from CF patients have decreased SERCA2 [10]. SERCA2 inhibition in airway epithelial cells also caused enhanced cell death upon ozone exposure [10]. Therefore, in this study, we systematically investigated ozone toxicity using polarized cultures of non-CF (16HBEo-) and CF (CF41o- and CF45o-) cells. We examined several components of cellular toxicity to assess the effect of ozone exposure.

Exposure to ozone (200 ppb, 8 h) caused increased monolayer disruption in CF cells (CF41o- and CF45o-) when compared to the non-CF controls (16HBEo-). Monolayer disruption in polarized cultures was measured by loss of trans-epithelial resistance (TER) as shown in Table 2.

**Table 2:** Effect of ozone exposure on transepithelial resistance (TER) in non-CF and CF airway epithelial cells.

Cell line	TER (Mean±SEM) Ohms.cm^2^0 ppb	TER (Mean±SEM) Ohms.cm^2^200 ppb
16HBEo-	522±06	500±45
CF41o-	369±23	190±68* #
CF45o-	394±33	251±41* #

*Indicates significant difference from 0 ppb, p<0.05.

# Indicates significant difference from 200 ppb non-CF, p<0.05.

**Figure 1:**
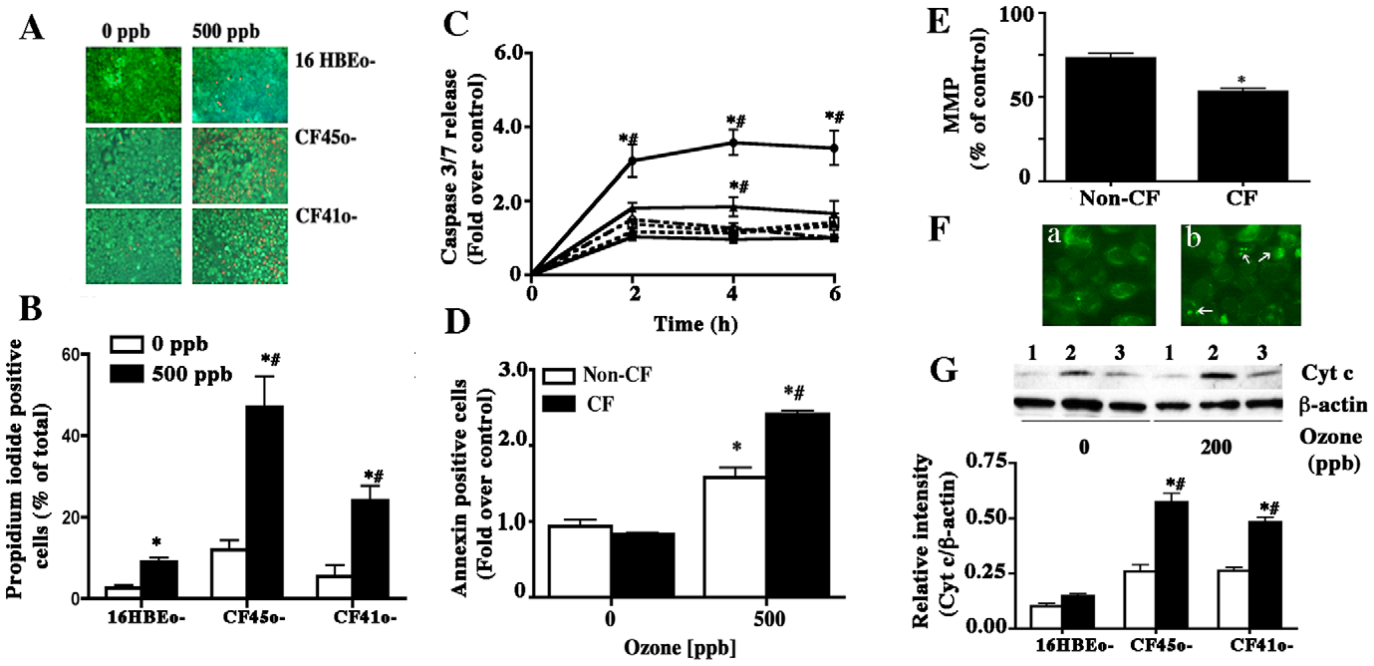
Ozone-induced toxicity in non-CF and CF airway epithelial cells. Polarized cultures of non-CF (16 HBEo-) and CF (CF41o- and CF45o-) cells were exposed to either 0 or 500 ppb ozone for 8 h. At the end of exposure cells were stained with Calcein AM (green, live) and propidium iodide (PI) (red, dead) (A). Quantitation of dead PI positive cells was performed using Image-Pro Plus version 4.0 (Media Cybernetics, Silver Spring, MD) and shown in (B). The percentage of dead cells was determined as the percentage of adherent positive cells relative to total number of adherent cells per ten high-power fields (x20). The assessment was performed blindfolded by two different individuals and the data obtained was analyzed by a statistician. The data shown are mean±SEM (n = 3). The image is representative of three independent experiments. * Indicates significant difference from 0 ppb control and # indicates significant difference from 500 ppb exposed non-CF, p<0.05. (C) Polarized cultures of 16HBEo- (-□-) and CF41o- (-▵-) and CF45o- (-○-) cells were exposed to either 0 (open symbols) or 500 ppb (closed symbols) ozone for 2, 4 or 6 h. At the end of each exposure supernatant media was collected and analyzed for caspase 3/7 release as described in the Methods. The data shown are mean±SEM (n = 3). * Indicates significant difference from 0 ppb control, and # indicates significant difference from 500 ppb exposed non-CF p<0.05. (D) Polarized cultures of non-CF 16HBEo- (white columns) and CF CF41o- (black columns) cells were exposed to either 0 or 500 ppb ozone for 8 h following which cells were collected and Annexin binding assay was performed as described in the Methods. Data is from one representative of 2 experiments. The data shown are mean±SEM (n = 6). The image is representative of two independent experiments. * Indicates significant difference from 0 ppb control and # indicates significant difference from 500 ppb exposed 16HBEo-. Cells p<0.05 (E) 16HBEo- and CF41o- cells were cultured on fibronectin-coated 6-well plates and exposed to ozone on the 4^th^ day of plating. Measurement of chloromethyltetramethylrosamine (MitoTracker Orange, Molecular Probes) fluorescence, an indicator of mitochondrial membrane potential (MMP), was performed as described in the Methods. A mean fluorescence intensity (MFI) of cells exposed to 0 ppb ozone was taken as control value. The data shown are mean±SEM (n = 6) The image is representative of two independent experiments. * Indicates significant difference from non-CF cells, p<0.05. (F) Ozone-mediated cytochrome c release in non-CF and CF cells. 16HBEo- and CF41o- cells were cultured on glass coverslips in 6-well plates and exposed to ozone (200 ppb) for 4 h. Cells were fixed and processed for cytochrome c releases using immunocytochemistry as described in the text. Representative images of (a) 16HBEo- and (b) CF41o- cells exposed to 200 ppb ozone are shown. The arrows indicate staining of cytochrome c. (G) Western blot of cytochrome c (‘Cyt c’) in the cytosolic fraction of 0 ppb and 200 ppb (4 h) ozone-exposed 16HBE (1), CF41o- (2) and CF45o- (3) cells. Image is representative of three independent experiments. The lower panel is a bar graph showing quantitation of cytochrome c Western bands. The data shown are mean±SEM (n = 3). * Indicates significant difference from 0 ppb control, and # indicates significant difference from 200 ppb (closed bars) exposed non-CF, p<0.05.

Measurements of uptake of Calcein-AM (live cell) and propidium iodide (dead cell) were also performed in polarized cultures of non-CF and CF cell lines. Propidium iodide uptake by these cells at 8 h of 200 ppb ozone exposure was minimal and could not be quantified using this method. Therefore, we exposed the cells at 500 ppb. Exposure of CF cells (CF41o- and CF45o-) to ozone (500 ppb) caused increased uptake of propidium iodide, further reflecting membrane damage and cell death (Figure 1 A & B). Caspase 3/7 release by non-CF and CF cells exposed to ozone (500 ppb) was also measured. Exposure to ozone caused an increase in caspase release in CF45o- cells at 2 h and was sustained until 6 h of exposure time. CF41o- cells also exhibited an increased caspase release at 4 h of exposure to ozone (Figure 1C). To further, validate and assess mode of cell death, cells were harvested by trypsinization and apoptosis was quantified using the Annexin V-binding method. Exposure to ozone (500 ppb, 8 h) of non-CF (16HBEo-) cells caused an increase in Annexin V-binding, which was significantly greater than that in 0 ppb exposed cells. Ozone exposure of CF cells (CF41o-) caused approximately two-fold increased annexin binding which was also significantly greater than in non-CF (16HBEo-) cells (Figure 1D). Additionally we performed TUNEL staining on the ozone exposed non-CF and CF airway epithelial cell cultures. Ozone exposure caused increased TUNEL positive cells in non-CF and CF airway epithelial cells (Figure S1). CF airway epithelial cells demonstrated enhanced TUNEL positivity as compared to the non-CF cells.

CF airway epithelial cells have decreased SERCA2 expression that could modulate intracellular calcium homeostasis [10], [25]. Both these effects could cause enhanced mitochondrial calcium uptake and that, upon further exposure to oxidant stress, could contribute to mitochondrial dysfunction and eventual cell damage [25], [26], [27]. We determined mitochondrial membrane potential (MMP) and cytochrome c release to assess mitochondrial function upon exposure to ozone in non-CF and CF cells cultured on collagen coated 6-well plates. Since these experiment were performed on cells cultured on collagen coated plastic dishes or glass coverslips, lower 200 ppb ozone exposure was sufficient to demonstrate its effect. Ozone exposure caused a small but nonsignificant decrease in MMP of non-CF (16HBEo-) cells. However, in CF (CF41o-) cells, there was an approximately 40% decrease in MMP after exposure to 200 ppb ozone as compared to 0 ppb ozone exposure. This was also significantly different from non-CF cells exposed to ozone at 200 ppb (Figure 1E). Similarly, we observed enhanced cytochrome c release (immunostaining of cells cultured on collagen-coated coverslips and by Western blots) in CF cells upon ozone exposure (Figure 1F & 1G).

### Ozone-induced enhanced cytokine release in CF cell lines

**Figure 2:**
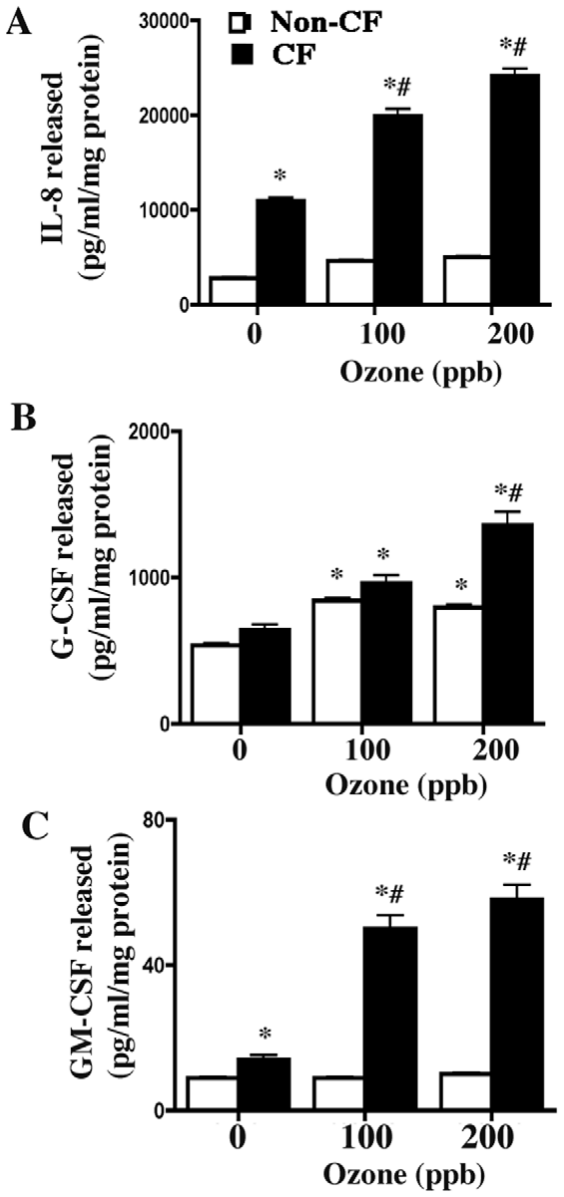
Ozone-mediated cytokine release in polarized cultures of non-CF and CF cell lines. 16HBEo- (white column) and CF41o- (black column) cells were cultured on collagen-coated 30 mm inserts. Once cells were polarized (8-10 days in culture) cells were exposed to 0, 100 or 200 ppb ozone with 200 µl media on the apical surface. At the end of exposure (18 h) an additional 200 µl media was added to the apical surface. Aliquots were collected after 4 h and analyzed for IL-8 (A), G-CSF (B) or GM-CSF (C) as described. The data shown are mean±SEM (n = 6). The image is representative of four independent experiments. * Indicates significant difference from 0 ppb control and # indicates significant difference from 100 or 200 ppb exposed non-CF p<0.05.

Ambient concentrations of ozone induce airway inflammation through release of proinflammatory mediators from airway epithelial cells [28]. We studied three cytokines viz. IL-8, G-CSF and GM-CSF in both apical and basalolateral compartments of polarized cultures. These cytokines are induced by ozone in bronchial epithelial cells and their levels are further enhanced upon ozone exposure in patients with other airway diseases [28], [29]. Exposure to ozone (100 or 200 ppb, 18 h) caused enhanced IL-8, GM-CSF and G-CSF release in the apical media of CF cell line CF41o- as compared to the non-CF 16HBEo- (Figure 2, A-C). We did not observe measurable cytokine content in basolateral media of these cells.

### Ozone-dependent cytokine release in differentiated primary non-CF and CF epithelial cells

**Figure 3:**
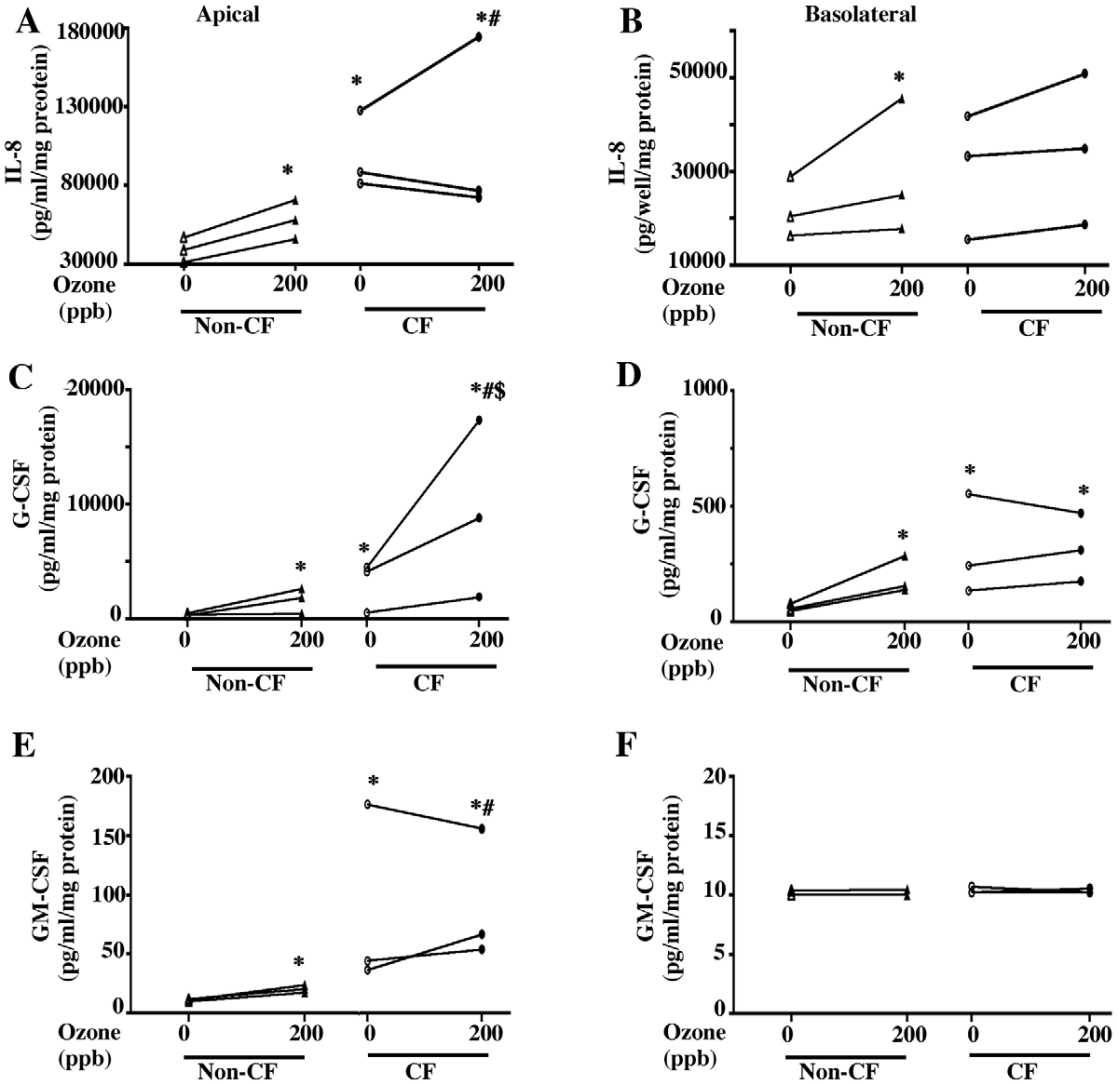
Effect of ozone exposure on the release of proinflammatory cytokines in differentiated cultures of non-CF (▵, 0 ppb and ▴, 200 ppb) and CF (○, 0 ppb and •, 200 ppb) primary airway epithelial cells. Cells were cultured on collagen-coated snapwells and allowed to grow and differentiate for 30 days. Before ozone exposure 100 µl media was added to the apical surface. At the end of exposure (18 h) an additional 200 µl media was added apically. After 4 h the media from apical (A) and basolateral (B) surface was collected and 100 µl of 6 mM DTT in PBS was added on the apical surface. The cells were incubated for 15 min in DTT and the supernatant fluid was carefully collected using a 1.0 ml pipette tip. The media and DTT solubilized mucin were analyzed for cytokines (IL-8 in A & B, G-CSF in C & D and GM-CSF in E & F) as described in the Methods. For apical cytokine content media and DTT solubilized mucin samples were pooled. Each symbol represents the mean of each donor. Three separate experiments were performed each time using one non-CF (donor 1-3) and one CF (4-6) donor. * Indicates significant difference from 0 ppb exposed non-CF, p<0.05 and # indicates significant difference from 200 ppb non-CF, p<0.05 and $ indicates significant difference from 0 ppb CF, p<0.05.

Air liquid interface (ALI) cultures of human nasal epithelial cells have been shown to release IL-8 upon exposure to ozone [30]. Differentiated primary airway epithelial cells (NHBE cells from a single donor) grown in co-culture with airway smooth muscle cells also release cytokines like IL-6 upon ozone exposure, whereas, the monolayer cultures do not release IL-6 upon exposure to ozone [7]. Studies comparing responses of differentiated primary airway epithelial cells derived from normal and diseased donors are lacking. We extended our study of ozone-mediated toxicity in non-CF and CF airway epithelium by using differentiated cultures of cells obtained from 3 non-CF and 3 CF donors (demographics shown in Table 1) to investigate the proinflammatory cytokine release response to ozone in close to *in vivo* airway epithelial cell models. These studies were performed in 30-day-old fully differentiated airway epithelial cell cultures details of which are described before [10]. We conducted these experiments at a relevant ambient concentration of ozone (200 ppb) and the exposure duration of 18 h. Proinflammatory cytokine production by epithelial cells from individual donors varied considerably. Our initial results with cells from 9 non-CF and 9 CF donors revealed even greater heterogeneity (data not shown). The differentiated cultures of airway epithelial cells secrete large amounts of mucus that may impact the cytokine measurement either by binding them or by limiting ozone access to cell surfaces [31]. Therefore, we compared apical IL-8 harvested by adding media to the apical surface to IL-8 harvested by further dissolution of apical mucus with DTT (Figure S2A). (It is important to note that even with DTT solubilization there was still a considerable amount of mucus gel remaining adherent which was impossible to completely remove without damaging cells.) We observed that the apical yield of solubilized IL-8 released from mucus was ∼40% greater when combined with the apical media. We used several controls shown in Figure S2A to ensure the authenticity of the assay including (1) 25 nM IL-8 standard in PBS or DTT (column 1 & 2 of Figure S2A) to demonstrate effect of DTT (2) The reaction was performed with prior incubation with IL-8 antibody or without the primary antibody in the sandwich ELISA to demonstrate the specificity of the reaction. (3) Further, purified porcine mucin was included to rule out the potential contribution of mucins directly to a positive reaction in the ELISA (column 5, Figure S2A). (4) IL-8 was added to porcine mucin to demonstrate potential interference of the mucin in the assay (column 6, Figure S2A). The results demonstrate that considerable IL-8 was retained within mucus. We did not detect mucin interference in the assay. The mucin content was also confirmed for these two fractions (Figure S2B, columns 3 & 4). Ozone exposure can cause enhanced mucin secretion in the upper airways of animal models [32], [33]. CF airway epithelial cells might have further enhanced mucin secretion. We estimated the apical media mucin content, as well as the solubilized mucin content from the apical surface of ozone-exposed differentiated cultures of non-CF and CF airway epithelial cells (Figure S2C). CF cells had increased mucin secretion relative to non-CF cells. In addition, mucin secretion was further enhanced upon ozone exposure in CF cells (Figure S2C). Therefore, we estimated the total IL-8 content (media+DTT solubilized mucin) harvested from the apical surface of differentiated non-CF and CF airway epithelial cells upon exposure to ozone. The IL-8 content of the basolateral media was also estimated. The overall pattern indicated that IL-8 and G-CSF were present in both apical and basolateral media (Figure 3A, B, C & D), whereas GM-CSF (Figure 3F) was barely detectable in the basolateral compartment. Ozone exposure caused enhanced proinflammatory cytokine (IL-8, G-CSF and GM-CSF) production at the apical surface in non-CF airway epithelial cells. The basolateral IL-8 and G-CSF contents were also significantly increased in the non-CF group upon ozone exposure. There was a significantly increased content of IL-8, G-CSF and GM-CSF on apical surface of 0 ppb exposed cultures of airway epithelial cells from CF donors as compared to the non-CF cultures. However, ozone exposure (200 ppb) did not further alter cytokine levels as compared to 0 ppb in a statistically significant manner in CF cells. The proinflammatory cytokine content of apical surface of 200 ppb ozone-exposed CF cells was still significantly greater than apical cytokine content of 200 ppb ozone-exposed non-CF airway epithelial cells.

### SERCA2 modifies ozone-induced cytokine release

**Figure 4:**
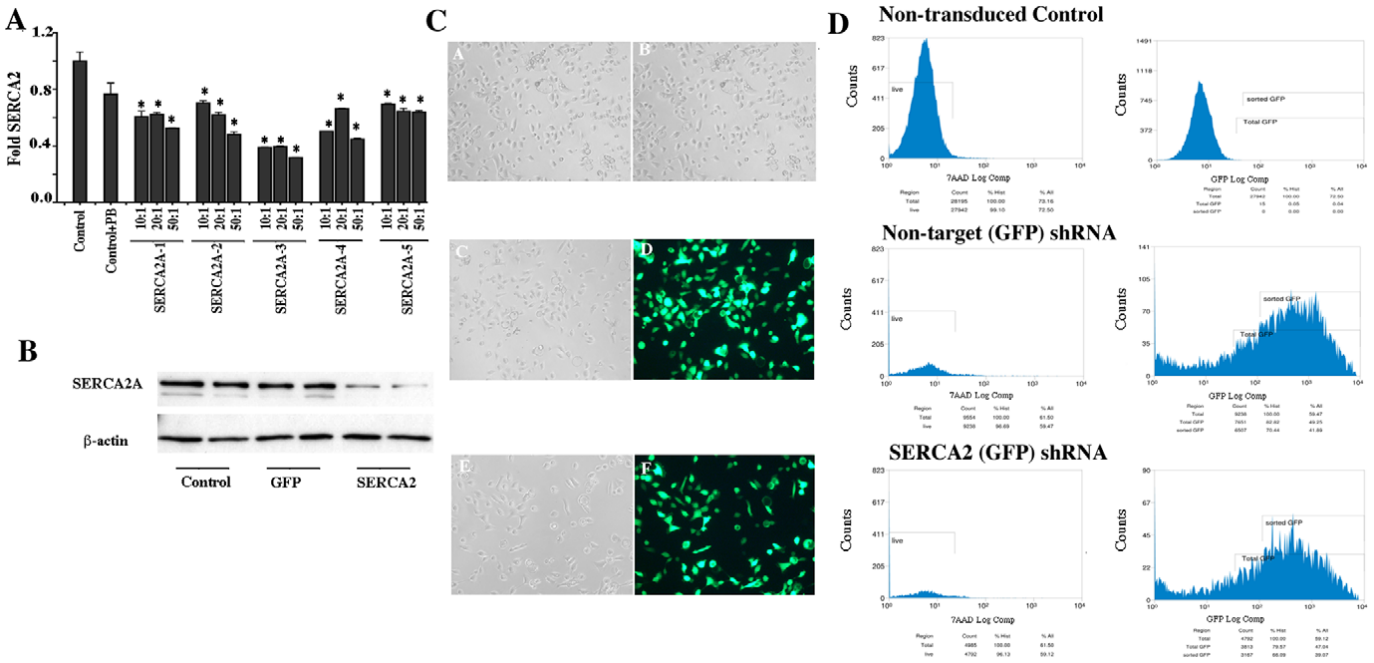
Lentiviral shRNA-mediated SERCA2 knockdown and its effect on cell phenotype and viability. A) Primary airway epithelial cells were cultured on collagen-coated 24-well plates. Replication-deficient lentivirus each carrying 5 different SERCA2 target shRNA (pLKO.1-CMV-tGFP-ATP2A2) (SERCA2A-1-5) were transduced at different MOI according to manufacturer's instructions and SERCA2 knock down was tested using real time RTPCR. The data shown are mean±SEM (n = 3) *p<0.05, significant difference between cells treated with SERCA2 shRNA and control. B) Primary airway epithelial cells were cultured on collagen-coated 6-well plates and transduced with SERCA2 shRNA (pLKO.1-CMV-tGFP-ATP2A2) or pLKO.1-CMVtGFP-TurboGFP control. Forty-eight hour post transduction cell lysates were prepared and SERCA2 Western blot was performed. C) Airway epithelial cell phenotype with and GFP expression by control and SERCA2 shRNA transduced cells. Cultured cells retained the morphology of airway epithelial cells (Brightfield light microscopy of untransduced, A & B, transduced C & E) and expressed GFP (Fluorescence microscopy of transduced D & F, matching area to the Brightfield in C & E). D) Flow cytometry of control, SERCA2 shRNA (pLKO.1-CMV-tGFP-ATP2A2) or pLKO.1-CMVtGFP-TurboGFP control cells with 7-AAD a cell death marker.

**Figure 5:**
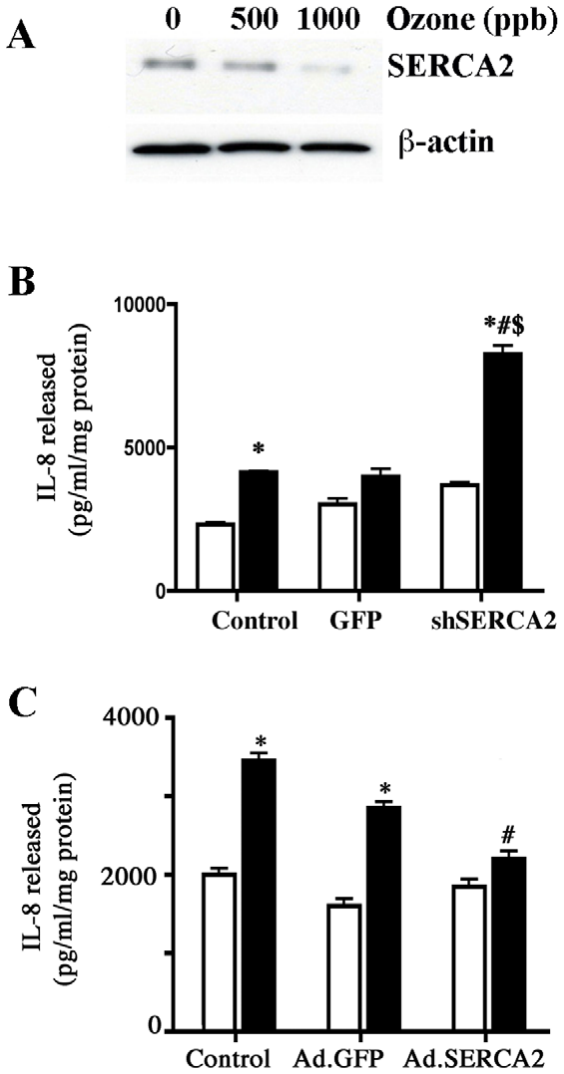
SERCA2 regulates ozone-mediated cytokine release by primary airway epithelial cells. Cells were cultured on collagen-coated snapwells at air-liquid interface (ALI) or on collagen-coated 6-well plates. (A) Primary airway epithelial cells were cultured at ALI and exposed to 500 or 1000 ppb ozone. At the end of exposure (4 h), lysates were prepared and SERCA2 protein was analyzed using Western blot. A representative blot from cells of one donor is shown. (B) Cells transduced with lentiviral GFP control or lentiviral GFP-SERCA2 shRNA were cultured on collagen-coated snapwells at air-liquid interface (ALI) and exposed to 0 (white column) or 200 (black column) ppb ozone as described in legend to Figure 3. Apical media was collected and analyzed. The data shown are mean±SEM (n = 6). The image is representative of two independent experiments. * Indicates significant difference from 0 ppb control and # indicates significant difference from 200 ppb exposed lentiviral GFP transduced cells, and $ indicates significant difference from 0 ppb exposed lentiviral GFP-SERCA2 shRNA transduced cells p<0.05. C) Primary airway epithelial cells cultured on 6-well plates were transduced with Ad.GFP or Ad.SERCA2. Exposure to ozone, 0 (white column) or 200 (black column) ppb for 18 h was carried out 48 h post transduction. The data shown are mean±SEM (n = 6). The image is representative of two independent experiments. * Indicates significant difference from 0 ppb and # indicates significant difference from 200 ppb ozone-exposed Ad.GFP transduced cells p<0.05.

A number of studies have implicated intracellular calcium mobilization in the activation of NF-κB and inflammatory signaling responses [15], [34]. SERCAs are responsible for (re) loading of endoplasmic reticulum calcium after such signaling events. Our previous work has established that CF airway epithelial cells have diminished SERCA2 expression [10]. We have also shown that siRNA mediated knockdown of SERCA2 in airway epithelial cells cultured on collagen-coated plastic dishes, causes enhanced cell death due to exposure to ozone, hydrogen peroxide and TNF [10]. Ozone exposure may itself modify SERCA2 expression in primary airway epithelial cells. Therefore, we investigated whether SERCA2 modulation by shRNA-mediated knockdown would affect ozone-induced proinflammatory cytokine release responses of polarized ALI cultures of primary airway epithelial cells. As shown in Figure 4A, using 5 different SERCA2 shRNA targets, we obtained a maximum knockdown using SERCA2 shRNA3 at 1∶50 MOI as measured by quantitative real-time RTPCR. Using this shRNA target we also demonstrated a knockdown in SERCA2 protein by western blot (Figure 4B). To observe the effect of SERCA2 knockdown on airway epithelial cell morphology, we cultured control and shRNA-transduced cells on collagen-coated dishes. Cellular morphology was not effected by SERCA2 knockdown as seen in Figure 4C. Based on GFP expression, the SERCA2 shRNA-transduced cells were sorted to purify them from untransduced cells (Figure 4D). Using 7-actinomycin D (7-AAD) dye to identify and exclude dead cells, we also demonstrated that degree of cell death was not increased in SERCA2 shRNA-transduced cells relative to GFP-transduced cells. Thus, SERCA2-shRNA transduction was not toxic (Figure 4D). Ozone exposure of undifferentiated ALI cultures of primary non-CF airway epithelial cells decreased expression of SERCA2 protein (Figure 5A). Short term (10 days) undifferentiated ALI cultures of primary airway epithelial cells expressing lentiviral SERCA2 shRNA were exposed to ozone (200 ppb), and apical media was analyzed for IL-8 release after exposure. Ozone exposure caused increased IL-8 release in these cultures. Specific inhibition of SERCA2 expression caused a further significant increase in IL-8 release upon ozone exposure (Figure 5B). In a complementary approach, we investigated whether SERCA2 overexpression would affect IL-8 release. In primary airway epithelial cells transduced with Ad.SERCA2, IL-8 release due to ozone was significantly decreased as compared to cells expressing Ad.GFP or with untransduced controls (Figure 5C) indicating that SERCA2 overexpression downregulates IL-8 production.

### Ozone-induced cytokine release in CF cell lines is NF-κB-mediated

**Figure 6:**
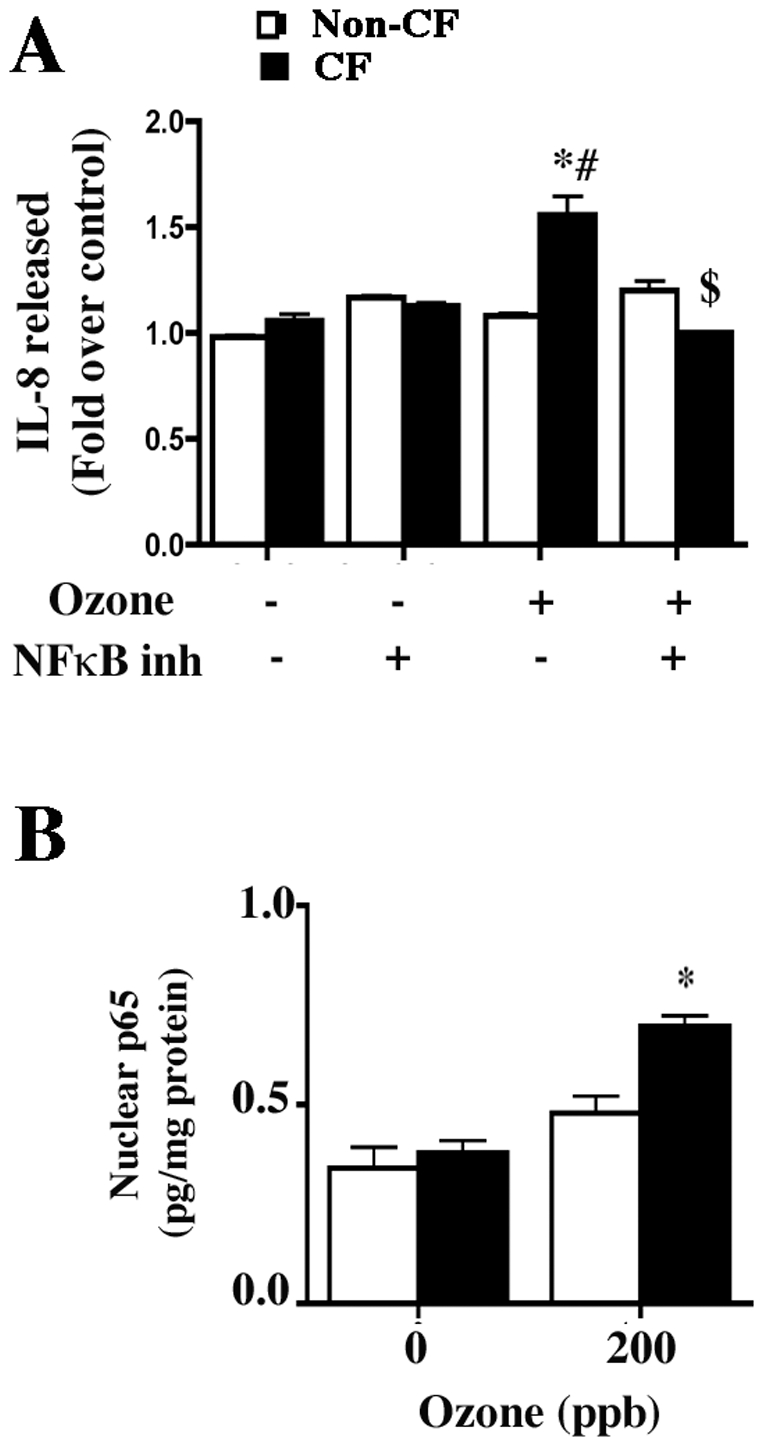
Ozone-induced cytokine release is NF-κB dependent. A) Effect of preincubation of cells cultured on collagen coated 6-well plates for 30 min with 10 µM NF-κB inhibitor [6-amino-4- (4-phenoxyphenylethylamino) quinazoline] or diluent on IL-8 release. The data shown are mean±SEM (n = 3). The image is representative of four independent experiments. * Indicates significant difference from 0 ppb exposed cells, # indicates significant difference from non-CF and $ indicates significant difference from CF cells not treated with NF-κB inhibitor during ozone (200 ppb) exposure p<0.05. For analysis of NF-κB activation, nuclear p65 was measured in non-CF and CF cells cultured on collagen-coated 6-well plates as described in the Methods (B). Cells were harvested and nuclear lysates were prepared after exposure to 200 ppb ozone. The nuclear fractions were assessed for p65 using the method described. The data shown are mean±SEM (n = 6). The image is representative of two independent experiments. * Indicates significant difference from 200 ppb ozone-exposed non-CF cells p<0.05.

Studies to evaluate the role of NF-κB in ozone-mediated cytokine release were performed on submerged non-confluent, non-polarized cells cultured on fibronectin and collagen-coated 6-well plates. This ensured effective delivery of the inhibitor to the cells. Preincubation with NF-*κ*B inhibitor (10 µM [6-amino-4- (4-phenoxyphenylethylamino) quinazoline] (EMD Biosciences, La Jolla, CA) caused IL-8 levels in ozone-exposed (200 ppb) CF cell line CF41o- to decrease to control values (Figure 6A). In CF cells, there was enhanced ozone-mediated p65 mobilization as well (Figure 6B).

### SERCA2 knockdown enhances NF-κB activation and proinflammatory cytokine production

**Figure 7:**
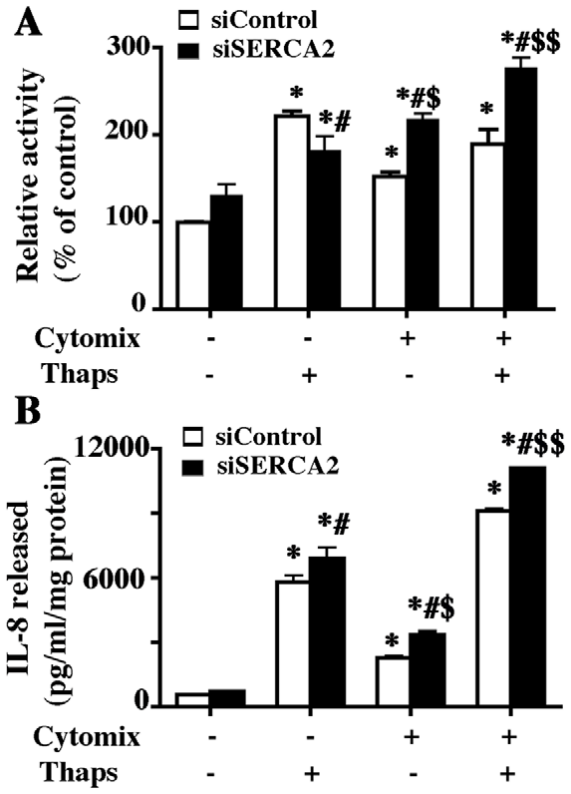
SERCA2 regulates NF-κB promoter activity and IL-8 release. NF-κB luciferase reporter-expressing 16HBEo- cells were cultured on collagen-coated 6-well plates and transfected with either control siRNA or SERCA2 siRNA. Forty-eight hour post transfection cells were treated with ‘Cytomix’ containing TNFα (20 ng/ml), IL-1β (10 ng/ml), IFNγ (10 ng/ml) and LPS (50 ng/ml) with or without thapsigargin (thaps; 2 µM)). (A) Cell lysates were prepared 24 h after treatment and luciferase activity was measured as described. The data shown are mean±SEM (n = 6). The image is representative of four independent experiments. * Indicates significant difference from untreated siControl transfected cells, # indicates significant difference from untreated siSERCA2 transfected cells, $ indicates significant difference from cytomix-treated siControl transfected cells, and $$ indicates significant difference from cytomix+thaps-treated siControl-transfected cells p<0.05. (B) Cells were cultured and treated as described above and supernatant media was collected and analyzed for IL-8. The data shown are mean±SEM (n = 3). The image is representative of two independent experiments. * Indicates significant difference from untreated siControl transfected cells, # indicates significant difference from untreated siSERCA2 transfected cells, $ indicates significant difference from cytomix-treated siControl transfected cells and $$ indicates significant difference from cytomix+thaps-treated siControl-transfected cells p<0.05.

The role of SERCA2 in modulating NF-κB activity was investigated using NF-κB-luciferase reporter gene expressing 16HBEo- cell lines. It was anticipated that inhibition of SERCA2 would induce NF-κB-driven luciferase activity. Inhibition of SERCA2 expression was performed using siRNA approach as described previously [10]. Thapsigargin was used to further decrease SERCA2 activity. Thapsigargin treatment by itself increased NF-κB-driven luciferase activity in both nonsilencing control and SERCA2-silenced cells. Treatment with cytomix (TNFα (20 ng/ml), IL-1β (10 ng/ml), IFγ (10 ng/ml) and LPS (50 ng/ml)) stimulated transcriptional activation of NF-κB that was greatest in SERCA2-silenced cells. Cytomix treatment in the presence of thapsigargin further increased NF-κB-driven luciferase activity in cells transfected with SERCA2-silencing siRNA (Figure 7A). SERCA2 inhibition caused increased release of IL-8, as shown by cytomix treatment of NF-κB-luciferase reporter gene expressing 16HBEo- cells (Figure 7B). Interestingly, IL-8 release by thapsigargin treatment alone exceeded IL-8 release by cytomix treatment in both siControl and siSERCA2 groups. Similarly, further increased IL-8 release by cytomix+thapsigargin treatment was observed in both groups, but IL-8 release in the SERCA2-silenced group was greater than in the nonsilenced group (Figure 7B).

### SERCA2 inhibition enhances ozone-induced NF-κB activity and IL-8 release

**Figure 8:**
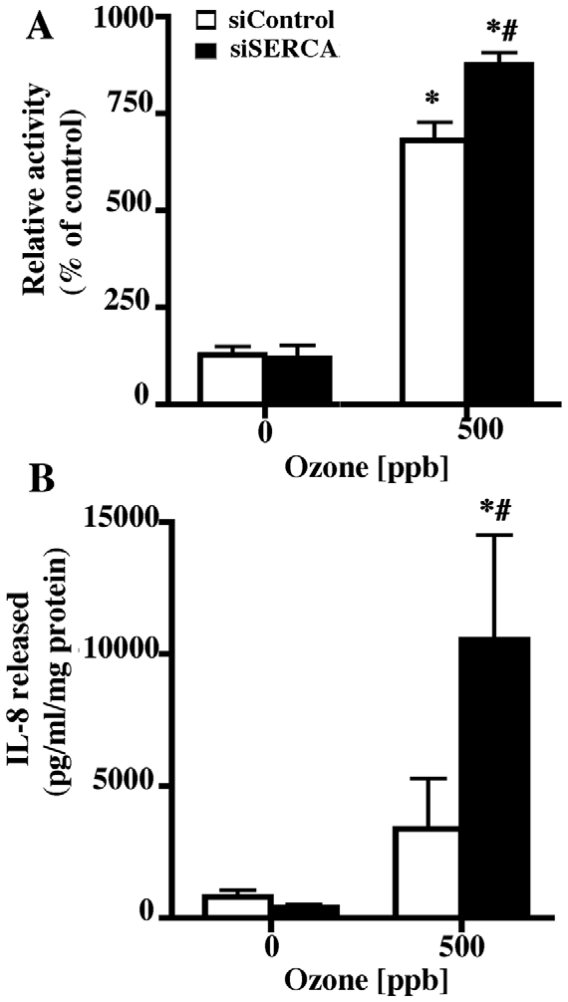
SERCA2 modulates ozone-induced NF-κB activity and IL-8 release. NF-κB luciferase reporter-expressing 16HBEo- cells were cultured on collagen-coated 6-well plates and transfected with either control siRNA or SERCA2 siRNA. Forty-eight hour post transfection cells were exposed to ozone (0 or 500 ppb). (A) Cell lysates were prepared after treatment and luciferase activity was measured as described. The data shown are mean±SEM (n = 6). The image is representative of two independent experiments. * Indicates significant difference from 0 ppb siControl transfected cells, # indicates significant difference from 500 ppb siControl transfected cells p<0.05. (B) Cells were cultured and treated as described above and supernatant media was collected and analyzed for IL-8. The data shown are mean±SEM (n = 6). The image is representative of two independent experiments. * Indicates significant difference from 0 ppb siControl transfected cells and # indicates significant difference from 500 ppb siControl transfected cells.

NF-κB-luciferase reporter gene expressing 16HBEo- cells were exposed to 0 or 500 ppb ozone. Ozone (500 ppb) exposure caused an increase in luciferase activity in both siControl and siSERCA2 transfected cells indicating transcriptional activation of NF-κB. However, at 500 ppb, the relative luciferase activity was greater in siSERCA2-silenced cells (Figure 8A). SERCA2 silencing also induced a greater IL-8 release response to ozone (500 ppb) exposure relative to siControl-transfected cells (Figure 8B). These results again indicate that SERCA2 can modulate NF-κB-dependent proinflammatory cytokine production.

### SERCA2 overexpression mitigates the enhanced proinflammatory response in CF cells

**Figure 9:**
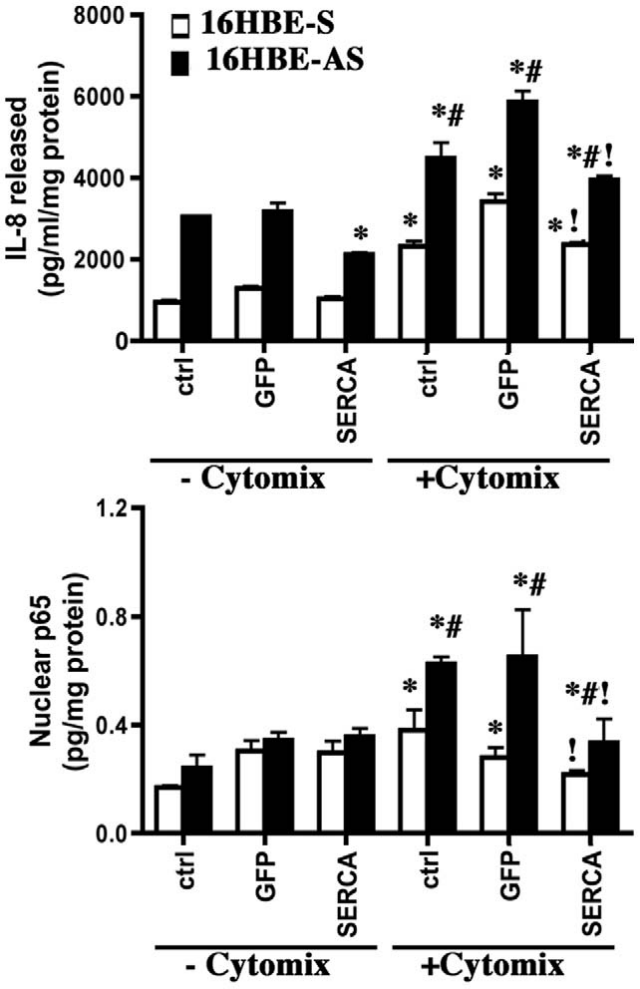
SERCA2 overexpression mitigates cytomix-induced enhanced IL-8 release and NF-κB activity in CF cells. 16HBEo- cell lines stably transfected with sense (16HBE-S, non-CF) and antisense (16HBE-AS, CF) CFTR oligonucleotide were cultured on 6-well plates as described in the Methods. On the 2^nd^ day of plating they were transduced with Ad.GFP or Ad. SERCA2 as described in the Methods. Cells were exposed to cytomix after 24 h incubation. Supernatant media was collected for IL-8 assay (A) and nuclear lysates were prepared for total p65 assay by ELISA. The data shown are mean±SEM (n = 6). * Indicates significant difference from untreated control cells, # indicates significant difference from cytomix treated non-CF and ! indicates significant difference from cytomix treated non-CF and CF cells p<0.05.

Previously, Kube et al. have shown that 16HBE-AS, a 16HBE cell line lacking the cystic fibrosis transmembrane conductance regulator activity, showed enhanced IL-8 and IL-6 responses compared with the control cell line 16HBE-S upon stimulation with *Pseudomonas aeruginosa* and a cytomix containing TNF-α and IL-1β [35]. This study further suggested that these cell lines might be useful for studying proinflammatory responses in CF airways [35]. We used these matched pair of non-CF and CF cell lines to study the effect of adenoviral-mediated SERCA2 overexpression (Figure S3) on cytomix-induced IL-8 production and NF-κB activity. Untreated control as well as GFP-transduced 16HBE-AS cells exhibited increased basal content of IL-8 that was significantly reduced by SERCA2 overexpression (Figure 9). IL-8 release measured 24 h post-treatment with cytomix was significantly increased in both control and CFTR-deficient cell lines. However, the cytomix-mediated IL-8 release in non-transduced control and GFP-transduced 16HBE-AS cells were significantly enhanced than the control cell line. Ad.SERCA2-transduction significantly abrogated enhanced IL-8 release by CF (16HBE-AS) cells and also diminished the response of control cells to cytomix (Figure 9A). Total p65 was also estimated in nuclear lysates of control and cytomix-treated 16HBE-AS and 16HBE-S cells transduced with Ad.GFP or Ad. SERCA2. Overexpression of SERCA2 also decreased cytomix-induced NF-κB activity, as evidenced by the decrease in total nuclear p65 in both control and CF cell lines (Figure 9B). These results further confirm that SERCA2 can modulate NF-κB activity and proinflammatory responses of airway epithelial cells.

## Discussion

Ozone is a highly reactive gas which, when present at higher concentrations, may cause airway damage via complex mechanisms. We have previously established the role of ozone-reactive surfactant phospholipids in modulating airway epithelial cell viability in response to ozone [8]. These derivatives not only caused apoptotic cell death but also induced proinflammatory responses that may potentiate airway injury in asthmatics and other susceptible patients like those with cystic fibrosis (CF). Increased oxidative stress and enhanced lipid-derived inflammatory mediators that are not modulated upon treatment is a characteristic of CF patient biofluids such as sputum during respiratory exacerbations [9]. A role for ozone in contributing to CF respiratory exacerbations has been suggested [1]. However, the type of injury and mechanisms involved in ozone-induced damage to CF airway epithelial cells is not clear. In this study, we investigated responses of CF and non-CF airway epithelium to ozone. We found that exposure of CF airway epithelial cells to ozone caused enhanced membrane damage, cell death and mitochondrial dysfunction. Further, polarized cultures of CF airway epithelial cell lines showed increased proinflammatory cytokine release upon ozone exposure. Similarly, exposure to ozone of differentiated airway epithelial cells from non-CF and CF donors yielded enhanced proinflammatory cytokine production. Ozone exposure decreased SERCA2, and SERCA2 knockdown caused an increased IL-8 release. Ozone-induced IL-8 release was reversed by SERCA2 overexpression. Ozone-induced IL-8 release was found to be NF-κB dependent. Interestingly, in cells with stable expression of the NF-κB luciferase reporter, SERCA inhibition caused increased NF-κB activity upon stimulation by cytomix containing IL-1β, TNFα, IFNγ and LPS. Cytomix and its components have been previously shown to induce NF-κB activity in airway epithelial cells including those of CF patients [36], [37], [38], [39], [40]. Increased NF-κB activity was also accompanied by enhanced IL-8 release in this system. A similar increase in NF-*κ*B activity and IL-8 release was observed upon exposure to higher ozone concentrations in the NF-*κ*B reporter cell lines. Overexpression of SERCA2 by adenovirus-mediated gene delivery abrogated the enhanced proinflammatory response of CFTR deficient CF -like 16HBE-AS cell lines.

Proliferation of mucus (goblet) cells and hypersecretion of airway mucus are important characteristics of CF. Exposure to ozone causes changes in airway fluid secretion similar to CF in animal models of primates and rats [41]. Moreover, exposure to ozone and particulate air pollutants were associated with an increased risk of pulmonary exacerbations and a decline in lung function in CF, suggesting a role of environmental exposures in causing exacerbations [1]. Our findings of decreased SERCA2 expression in CF epithelium and enhanced susceptibility to oxidative stress such as that caused by ozone further suggests that survival responses of CF epithelium are compromised [10]. The fact that there was increased loss of transepithelial resistance upon exposure to ozone demonstrates greater susceptibility of CF airway epithelial cells to such stress. However, at lower ozone concentrations there was no quantifiable propidium uptake to demonstrate membrane damage and death. The loss of TER without cell death could be attributed to other possible mechanisms such as those that occur upon infection by viral particles [42]. Moreover, lifting of a single cell from a tight monolayer could also dramatically reduce TER. Further, our results of increased membrane damage and apoptosis in CF cell are consistent with our previous findings as well as those of other investigators wherein ozone or ozone-generated oxidized lipids caused apoptosis [8], [18], [43]. By contrast, acute exposure to higher ozone concentrations caused cell death via non-apoptotic mechanisms in airway epithelium in rats [44]. SERCA2 dysfunction would tend to cause chronically enhanced cytosolic calcium that could be harmful to cellular organelles including mitochondria and even predispose to cell death. Moreover, pre-existing oxidative stress in CF airway epithelium could lead to greater mitochondrial and cellular dysfunction upon exposure to additional oxidizing environmental pollutants such as ozone.

Elevated expression of proinflammatory cytokines appears to contribute to chronic airway disease exacerbations. Accordingly, ozone-induced bronchial epithelial cytokine expression was increased in mild allergic asthmatic subjects as compared to healthy subjects [29]. Those cytokines included, among others, IL-8 and GM-CSF. Decreased SERCA2 expression and increased proinflammatory cytokine production have been recently observed in airways and cells of asthma patients [45]. Our studies with polarized cultures of non-CF and CF airway epithelial cell lines and differentiated cultures of non-CF primary airway epithelial cells demonstrated enhanced stimulation of proinflammtory cytokine at their apical surface when exposed to ozone. The apical proinflammatory cytokine content, with and without ozone exposure, of differentiated primary airway epithelial cells from CF subjects was significantly greater than the cells from airways of non-CF subjects. The fact that significant effect of ozone exposure was not observed in differentiated CF airway epithelial cell cultures could be attributed to several factors, including but not limited to (1) these are differentiated cells, including mucus-secreting cells, and the cell surface is covered with a thick layer of mucus. Ozone is a highly reactive gas, one that likely does not penetrate the cell surface, or the layer covering the cell surface, by more than 0.1 micron. (2) CF cells in culture are covered with greater amounts of cell surface mucus than non-CF cells, and this increases greatly upon ozone exposure. Given that our ability to recover the mucus layer from each cell well was incomplete, we know that our estimate of IL-8 and likely other cytokines produced by each culture was an underestimate of the total cytokines produced, but, among these, this appeared to be proportionately greatest from cultures of CF cells. For this reason, we believe that the degree to which cytokine levels in CF cultures exceeded those present in the non-CF cultures is likely still greater than reported here. (3) We demonstrated that significant amounts of IL-8 are retained in the cell surface mucus layer. Despite the potential interference by cell mucus layer with ozone penetrance, there was an enhancement, albeit inconsistent, of G-CSF and IL-8 by CF primary cells in response to ozone. In addition, CF cells tended to have higher basal levels of all three cytokines assessed, even in absence of ozone exposure. Ozone itself caused decreased SERCA expression in non-CF primary human airway epithelial cells. SERCA expression was already decreased at baseline in the CF cells. This may be another factor contributing to the fact that inflammatory cytokine expression was not consistently increased by ozone in primary CF human airway epithelial cells. Clearly, donor variability also contributed. This may have been related to differences in degree of mucus cell differentiation in different cultures, or other factors. Donor variability in primary human airway epithelial cells has been addressed meticulously by Zabner et al., especially within the context of differentiated epithelial cell cultures from airways of CF subjects [46]. Limitations in cell culture systems may also affect cytokine production. For example, cultures of primary airway epithelial cells from CF patients immersed in culture medium (not at ALI) exhibited higher proinflammatory cytokine production than non-CF cells in response to bacterial pathogens, whereas ALI cultures did not do so in the same study [47], [48]. Further the differences from other studies may also be related to technical aspects of handling of the cells; specifically that our rocking exposure system needed for ozone and the potential impact of variable ATP release and cell surface fluid regulation and mediator concentrations in CF and non-CF cultures in the presence and absence of motion may have varied.

Our studies indicated that indeed exposure to ozone of cells with CFTR dysfunction might predispose to proinflammatory cytokine production in an NF-κB dependent manner. CFTR dysfunction-induced enhanced oxidative stress, decreased anti-inflammatory signaling and altered processing of IκB could result in increased cytokine production in CF cells [38], [49]. Ozone exposure by itself could cause downregulation of CFTR and decreased CFTR chloride current in human bronchial epithelial cells [50]. Further, a role of CFTR during the initiation of apoptosis in airway epithelial cells also has been proposed [51], [52]. We have shown previously that pharmacologic and genetic modulation of CFTR in airway epithelial cells decreases SERCA2. Thus CFTR dependent SERCA2 modulation seems to be an important regulator of airway epithelial cell responses to oxidative stressors such as ozone.

Thapsigargin (a specific SERCA inhibitor) causes enhanced bacterial infection, NF-κB activation and IL-8 release in airway epithelial cells in a cytosolic calcium dependent manner [16]. Although SERCA2 expression itself may be modified by cytokines [53], direct modulation of epithelial production of IL-8 by SERCA2 has not been previously described. Our findings suggest that decreased SERCA2 expression in CF airway epithelial cells contributes to the exaggerated proinflammatory response by modulating NF-κB. The exact mechanisms by which this occurs are potentially important but yet to be investigated. However these studies indicate that pharmacologic modulation of SERCA2 expression and/or activity could prove useful in limiting CF airway inflammation or controlling exacerbations due to environmental pollutants like ozone.

## Supporting Information

Figure S1 **Effect of ozone exposure on non-CF and CF airway epithelial cells.** Polarized cultures of non-CF, 16 HBE and CF, CF45o- and CF41o- cells were exposed to either 0 or 500 ppb ozone for 8 h. At the end of exposure cells were fixed and TUNEL staining was performed *in situ* as described in the Methods section. Panels A-C represents the controls of 16HBE, CF45o- and CF41o- respectively and panels D-F represent the 500 ppb exposed 16HBE, CF45o- and CF41o- cells. Arrows indicate positive TUNEL stained cells. Inset of E shows image focused on positive stained cells.
(TIF)

Figure S2 **Retention of cytokines in primary airway epithelial cell surface mucin secretions.** Primary airway epithelial cells were cultured on collagen-coated inserts. Cells were maintained at air-liquid-interface (ALI) upon polarization and differentiated for 4 weeks. Cell surface was washed with PBS and 200 µl ALI media was added on the apical surface and cells were incubated for overnight. At the end of incubation apical media was collected. On another set 6 mM DTT containing PBS was added and cells were incubated for 15 min and then apical solution was collected to harvest the mucins. Both type of apical fluid were analyzed for IL-8 (panel A) or respiratory mucins (panel B). Data shown is mean±SEM (n = 6). *Indicates significant difference from “media” content p<0.05. Panel C demonstrates the mucin content of non-CF and CF cells upon ozone exposure. Primary non-CF and CF airway epithelial cells from 3 donors each were cultured and exposed to ozone as described in legends to Figure 3. The supernatant media was harvested and DTT (6 mM) containing PBS was added on the apical surface. After 15 min the apical fluid was harvested carefully. Data shown is mean±SEM (n = 9). * Indicates significant difference from 0 ppb non-CF and # indicates significant difference from 200 ppb exposed non-CF cells p<0.05.
(TIF)

Figure S3 **SERCA2 overexpression by adenovirus-mediated gene transfer.** Primary airway epithelial cells were cultured on collagen-coated dishes and recombinant viruses were added to cell cultures (multiplicity of infection, MOI 10∶1) on day 3 of culture. Transduction efficiency was estimated by observing green fluorescence of adenoviral GFP-transduced cells. Cell lysates were prepared and analyzed for SERCA2 protein expression by Western. The data shown are mean±SEM (n = 3) * Indicates significant difference between cells treated with Ad.SERCA2 and control (untreated or Ad.GFP treated) p<0.05.
(TIF)
