# Supplementary figures and images for: SERCA2 Regulates Non-CF and CF Airway Epithelial Cell Response to Ozone
Source: PLoS One. 2011 Nov 11;6(11):e27451. doi: 10.1371/journal.pone.0027451 (PMC3214057; doi:10.1371/journal.pone.0027451)

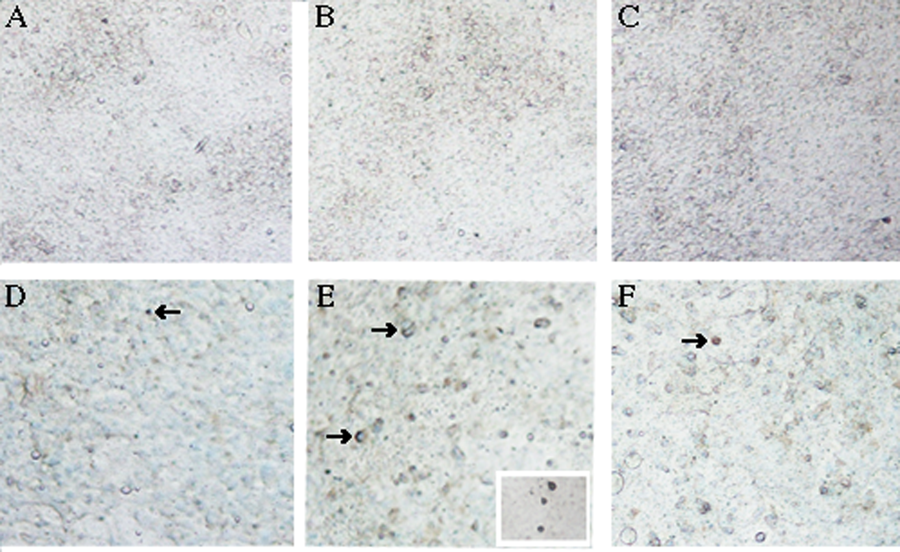

Supplement: Figure S1 — Effect of ozone exposure on non-CF and CF airway epithelial cells. Polarized cultures of non-CF, 16 HBE and CF, CF45o- and CF41o- cells were exposed to either 0 or 500 ppb ozone for 8 h. At the end of exposure cells were fixed and TUNEL staining was performed in situ as described in the Methods section. Panels A-C represents the controls of 16HBE, CF45o- and CF41o- respectively and panels D-F represent the 500 ppb exposed 16HBE, CF45o- and CF41o- cells. Arrows indicate positive TUNEL stained cells. Inset of E shows image focused on positive stained cells. (TIF) [file pone.0027451.s001.tif]

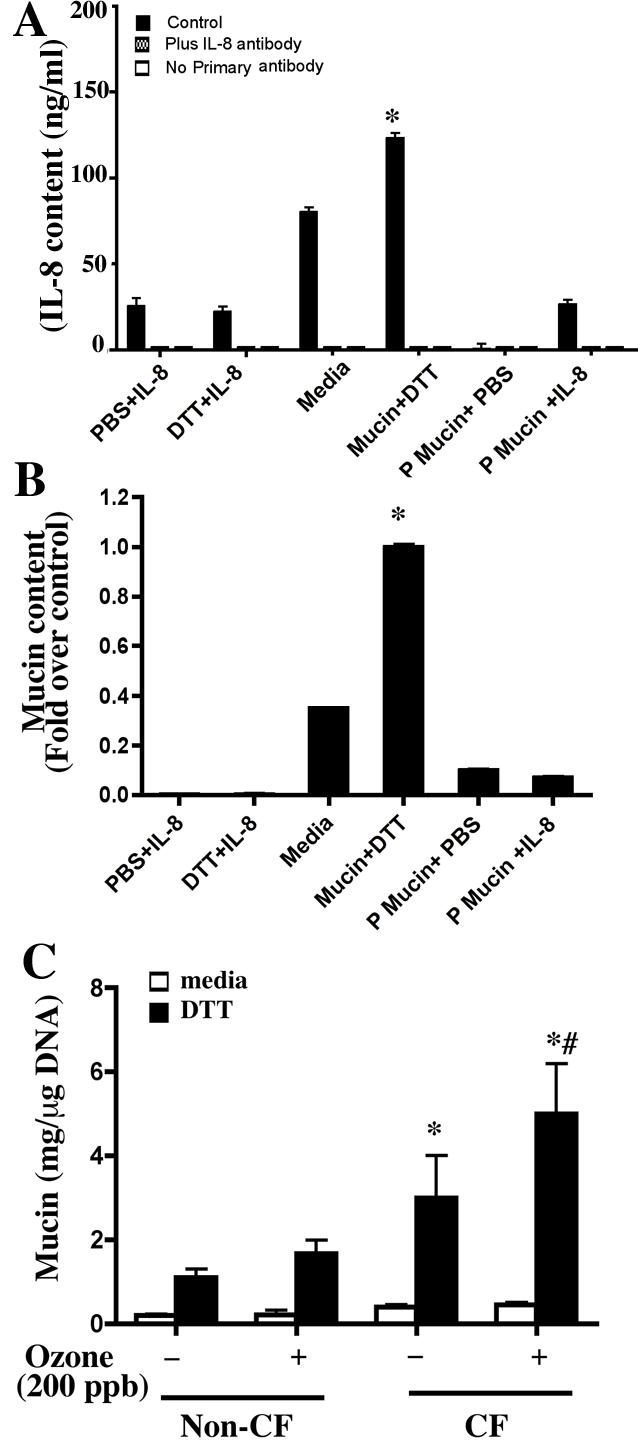

Supplement: Figure S2 — Retention of cytokines in primary airway epithelial cell surface mucin secretions. Primary airway epithelial cells were cultured on collagen-coated inserts. Cells were maintained at air-liquid-interface (ALI) upon polarization and differentiated for 4 weeks. Cell surface was washed with PBS and 200 µl ALI media was added on the apical surface and cells were incubated for overnight. At the end of incubation apical media was collected. On another set 6 mM DTT containing PBS was added and cells were incubated for 15 min and then apical solution was collected to harvest the mucins. Both type of apical fluid were analyzed for IL-8 (panel A) or respiratory mucins (panel B). Data shown is mean±SEM (n = 6). *Indicates significant difference from “media” content p<0.05. Panel C demonstrates the mucin content of non-CF and CF cells upon ozone exposure. Primary non-CF and CF airway epithelial cells from 3 donors each were cultured and exposed to ozone as described in legends to Figure 3. The supernatant media was harvested and DTT (6 mM) containing PBS was added on the apical surface. After 15 min the apical fluid was harvested carefully. Data shown is mean±SEM (n = 9). * Indicates significant difference from 0 ppb non-CF and # indicates significant difference from 200 ppb exposed non-CF cells p<0.05. (TIF) [file pone.0027451.s002.tif]

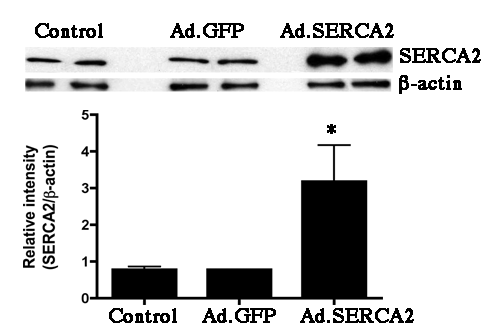

Supplement: Figure S3 — SERCA2 overexpression by adenovirus-mediated gene transfer. Primary airway epithelial cells were cultured on collagen-coated dishes and recombinant viruses were added to cell cultures (multiplicity of infection, MOI 10∶1) on day 3 of culture. Transduction efficiency was estimated by observing green fluorescence of adenoviral GFP-transduced cells. Cell lysates were prepared and analyzed for SERCA2 protein expression by Western. The data shown are mean±SEM (n = 3) * Indicates significant difference between cells treated with Ad.SERCA2 and control (untreated or Ad.GFP treated) p<0.05. (TIF) [file pone.0027451.s003.tif]
